# Periprosthetic Joint Infection Occurring Following a Femoral Subcutaneous Cyst: A Rare Complication Post–Total Knee Arthroplasty

**DOI:** 10.1155/cro/7710384

**Published:** 2025-04-24

**Authors:** Naohiro Oka, Shigeshi Mori, Yu Shinyashiki, Nobuhisa Shokaku, Kenji Yamazaki, Koji Goto, Daisuke Togawa

**Affiliations:** ^1^Department of Orthopedic Surgery, Kindai University Nara Hospital, Ikoma, Nara, Japan; ^2^Department of Orthopedic Surgery, Kindai University Faculty of Medicine, Osakasayama, Osaka, Japan

**Keywords:** femoral subcutaneous cyst, periprosthetic joint infection, total knee arthroplasty

## Abstract

Herein, we present a rare case of periprosthetic joint infection (PJI) which was triggered by an infection with a latent subcutaneous cyst on the thigh and occurred in a strange course following total knee arthroplasty (TKA). An 87-year-old female underwent right TKA followed by left TKA 5 months later. Six weeks after left TKA, a painful subcutaneous induration appeared in the left medial thigh. Magnetic resonance imaging revealed a 30∗50-mm multifocal mass. The cystic fluid was brown and cloudy, indicating an infected cyst. Oral antimicrobial therapy was initiated for 7 days. Nine weeks after the left TKA, a left calcaneal fracture occurred. Subsequently, edema of the lower extremities and pain in the left knee gradually developed. Arthrocentesis was performed twice: joint fluid Gram staining and culture examination were negative. However, at 12.5 weeks, an alpha-defensin test of the synovial fluid was positive. Therefore, PJI was diagnosed. DAIR was performed, followed by multiantibiotic therapy. The infection subsided gradually. Edema of the lower limbs was treated with oral diuretics, lymphatic massage, and compression stockings. Consequently, the lower limb edema also improved. In this case, infection of a latent subcutaneous cyst in the thigh occurred and spread around the prosthesis due to leg edema, which was associated with loss of lower limb motion due to a calcaneal fracture. The presence of a potential thigh subcutaneous cyst is a risk factor for PJI. Moreover, lower extremity edema occurs by decreasing lower extremity motion, such as after a calcaneal fracture, and it increases the risk of extending extra-articular infection to the PJI. Potential thigh subcutaneous cysts and lower extremity edema are risk factors for the development of PJI. Orthopedic surgeons need to be aware of these facts during follow-up after TKA.

## 1. Introduction

Total knee arthroplasty (TKA) is an excellent procedure for end-stage knee arthritis and is effective in relieving pain and improving knee function [1]. However, periprosthetic joint infection (PJI) is a serious complication that affects joint function and can lead to destructive outcomes. Therefore, prevention and early recognition of PJI signs are important for orthopedic surgeons performing TKA. Early intervention, such as debridement, antibiotics, irrigation, and retention (DAIR), is essential for treating acute PJI and can have a relatively good outcome [2]. However, the signs of infection are often inconsistent and difficult to diagnose.

Herein, we present a complicated case of acute PJI following TKA. The infection first occurred in a preexisting femoral subcutaneous cyst. An incidental calcaneal fracture subsequently occurred, and exacerbation of lower limb edema associated with decreased activity delayed the diagnosis of PJI. This report highlights the importance of managing adjacent soft tissue infections and postoperative lower extremity edema after TKA.

## 2. Report of the Case

An 87-year-old female patient visited our clinic with pain in both knees for a few years. At the clinic, she underwent a trial of conservative treatment, oral NSAIDs, or hyaluronic acid injections in the knee joint. However, the patient's symptoms did not improve. She was then referred to our hospital to undergo TKA. The patient had a history of stroke and was mildly obese with a body mass index (BMI) of 34. However, she had no other primary illnesses, such as diabetes.

**Figure 1:**
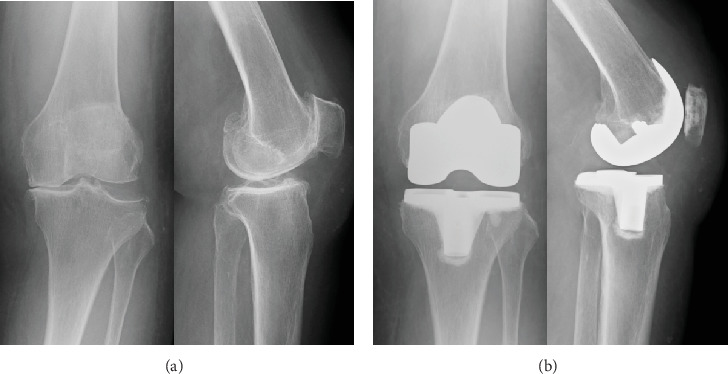
(a) Preoperative anteroposterior and lateral radiograph of the left knee. (b) Radiograph post-TKA.

The patient underwent right and left TKA 5 months later (Figure 1). No adverse intraoperative complications were observed. The patient recovered well and was discharged on Postoperative Day 19 following left TKA and Postoperative Day 26 following right TKA. The duration of hospitalization was slightly longer after the left TKA compared to the right TKA owing to the delayed healing of the surgical wound. The treatment was successful and the patient's postoperative course was uneventful with good knee function.

**Figure 2:**
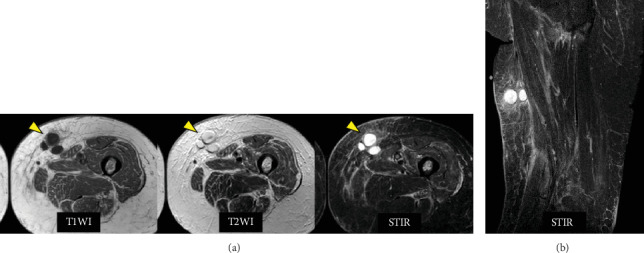
MRI images of femoral subcutaneous cysts. MRI of the left femur: (a) axial view and (b) coronal view. The subcutaneous cyst was located just beneath the sartorius muscle, approximately 18 cm distally from the pubic symphysis. The subcutaneous multifocal cyst (yellow arrow) showed diffuse T1, T2, and STIR hyperintensities. There was no inflammatory spillover around the cysts. This indicated that the mass was a nonmalignant tumor surrounded by a capsule.

However, 6 weeks after the left TKA, a painful subcutaneous induration appeared in the left medial thigh. Magnetic resonance imaging (MRI) revealed a 30 × 50-mm multifocal mass in the medial thigh, which had low intensity on T1-weighted images and high intensity on T2-weighted images (Figure 2). The inflammatory response was mildly positive (WBC: 5750/*μ*L, CRP: 2.9 mg/dL, ESR: 29 mm/h) regarding blood test parameters.

Cystic fluid obtained under ultrasonographic guidance was brown and cloudy. Bacterial culture revealed *Staphylococcus lugdunensis* (CNS; coagulase-negative staphylococci) in the puncture fluid. The cyst was considered to be infected, and the patient was started on oral antimicrobial therapy (levofloxacin hydrate, 500 mg/day). One week later (L-TKA, PO: 7 weeks), the inflammatory response decreased (WBC: 4960/*μ*L, CRP: 0.5 mg/dL, ESR: 29 mm/h), and antimicrobial medication was discontinued.

**Figure 3:**
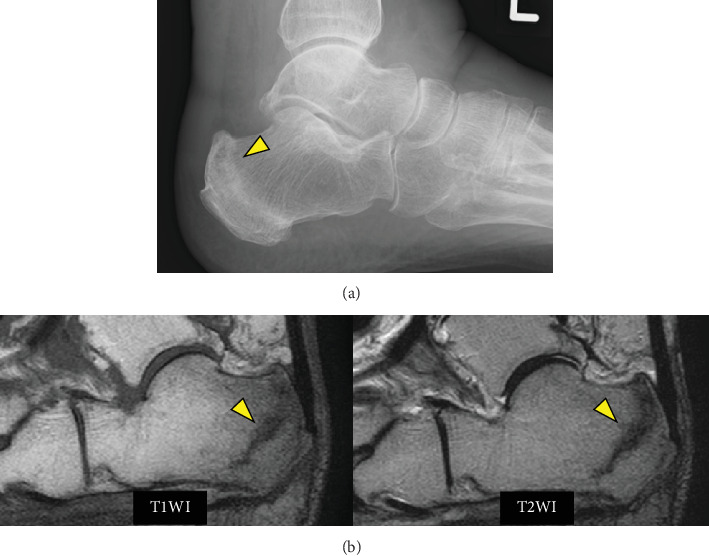
(a) Initial radiograph of the calcaneus taken 9 weeks following total knee arthroplasty, at symptom onset. The calcaneal posterior facet is displaced. (b) The sagittal view of the T1- and T2-weighted images shows an irregular fracture line in the calcaneus from the insertion of the plantar aponeurosis to the insertion of the Achilles tendon.

On Postoperative Week 9, the patient complained of left heel pain. Radiography and MRI revealed a calcaneal fracture (Figure 3). She had no obvious history of trauma, and we determined that this fracture was associated with a change in lower-extremity alignment due to TKA. We instructed the patient not to bear weight on her left lower limb to allow the fracture to heal. However, edema gradually developed in the left lower limb. Although she had no knee pain after the calcaneal fracture and no worsening lower edema, the left knee pain gradually worsened (approximately at PO: 12). Furthermore, at this time, the blood test showed a slight increase in inflammatory markers (WBC: 6840/*μ*L, CRP: 3.1 mg/dL, ESR: 33 mm/h).

**Figure 4:**
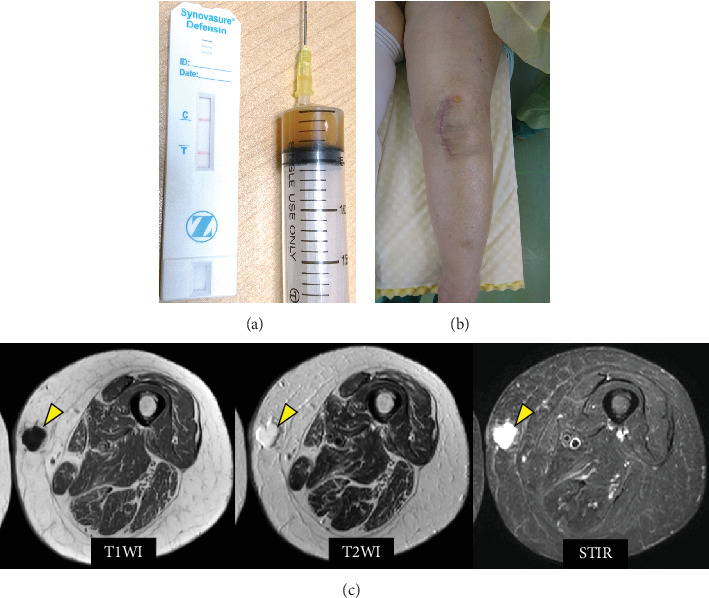
(a) Synovial fluid of the left knee showing yellow and mildly turbid fluid. The alpha-defensin test from this sample revealed positive lines. (b) The affected limb just prior to DAIR showing lower limb edema. (c) MRI of the thigh after the infection subsided. The cyst showing hypointense in T1WI, isointense in T2WI, and hyperintense in STIR. The subcutaneous cyst had shrunk (yellow arrow).

Arthrocentesis was performed twice, and joint fluid Gram staining and culture examination results were negative. However, the alpha-defensin test was positive, and a diagnosis of PJI was considered based on the criteria of the 2018 International Consensus Meeting on Musculoskeletal Infection [3]. We performed DAIR at 12.5 weeks postoperatively (Figure 4a,b). In particular, in open capsule synovectomy, we changed the polyethylene insert and placed joint drainage for 72 h after surgery. Methicillin-resistant coagulase-negative staphylococci (MR-CNS) were detected in the bacterial culture of a specimen obtained from the knee joint during surgery.

The patient received three different antibiotics (meropenem, teicoplanin, and rifampicin) and then was switched to linezolid and rifampicin. Two weeks after DAIR, the antibiotics were changed to oral drugs (rifampicin, clindamycin, and minocycline) and were de-escalated over 3 months. Antibiotic therapy was completed 3 months after DAIR, and the inflammatory response subsided. After the administration of antibiotics, the subcutaneous femoral cyst regressed, and pain decreased (Figure 4c).

Immediately after DAIR, treatment for lower limb edema was initiated. Despite research on the causes of edema, such as pelvic tumors or deep vein thrombosis, no such causes of edema were found in our patient. The patient received oral diuretics, lymphatic massage, and compression stockings for treatment of the lower edema. As a result, lower limb edema improved. The patient had no signs of recurrent PJI 2 years after DAIR. The patient also had good knee function (Table 1).

## 3. Discussion

TKA is a major treatment for knee osteoarthritis (OA) and is currently performed at a rate of 70,000 cases per year in Japan. TKA has been improving due to advancements in surgical techniques, implant materials, and designs [4, 5]. TKA is associated with several complications. Among them, PJI especially reduces knee function and decreases patients' ability to perform activities of daily living (ADL) of patients. Many causes of PJI and its pathophysiology have been reported [6]. This is the first report of PJI after TKA that was predicted to have developed from an infection of a subcutaneous cyst on the medial thigh on the operated side.

This report presents two important findings. The presence of an underlying subcutaneous cyst in the thigh is a potential cause of PJI after TKA. There are some reports that secondary swelling of the femoral inguinal lymph nodes occurs after PJI following TKA [7]. However, in this case, the first step in the development of PJI was a subcutaneous femoral cyst infection. To the best of our knowledge, this is the first report of PJI after TKA caused by a subcutaneous femoral cyst infection.

In the diagnostic process for this case, we initially considered several different possibilities as the source of the PJI. Infection spreading from a distant infectious focus, which is generally considered to be the source of PJI after TKA, has been observed previously. In rare cases of infection spreading from adjacent infected sites, such as descending spinal infection, pubic symphysitis or intestinal fistula has also been reported [8]. However, none of these were evident in this case. The only significant finding (i.e., causative bacteria were detected) as a source of infection was the femoral subcutaneous cyst.

MRI is the most effective method for diagnosing subcutaneous femoral cysts (including lymph nodes). Alternatively, ultrasonography serves as a simpler diagnostic method [9]. Some reports suggest that evaluation of inguinal lymph node size using ultrasonography is useful for assessing infection after TKA [10]. From a medical and economic perspective, it is impractical to check all patients undergoing TKA for femoral subcutaneous cysts using preoperative ultrasonography. However, we believe that it is important to check for the presence of subcutaneous cysts during the preoperative physical examination not only around the knee but also around the thigh (especially on the medial side) to prevent infection after TKA. Second, if measures are not taken to prevent the worsening of lower extremity edema during follow-up after TKA, there is a possibility of progression to PJI. Lower extremity edema is a common phenomenon that occurs not only with TKA but also after lower extremity fractures or surgeries [11, 12]. The causes of postoperative lower limb edema include abnormal venous return after surgery, lymphatic stasis, and DVT associated with decreased postoperative leg motion [13, 14]. If there is an infected cyst at a site other than the knee in the lower extremity after TKA and lower edema develops, there may be a secondary spillover of infection around the TKA.

Several mechanisms by which lower limb edema may contribute to infection worsening have been proposed. When lower limb edema occurs, venous and lymphatic return is obstructed [15]. Stagnation and reflux of lymph fluid and venous blood may develop into PJIs on the same side as a lower limb abscess, other than the prosthetic joint.

In this case, close examination for the presence of pelvic organs or DVT as a cause of lower extremity edema was not performed. The cause of lower extremity edema in this case may have been a calcaneal fracture. Calcaneal fractures are relatively common complications of TKA [16]. Calcaneal fractures may lead to decreased mobility, such as difficulty loading the affected limb, resulting in increased edema of the lower extremities. PJI may develop from infected subcutaneous cysts due to venous stasis in the lower extremities caused by leg edema. In our opinion, surgeons should carefully monitor for the occurrence of PJI when an event occurs that causes lower extremity edema, such as calcaneal fractures after TKA.

Some limitations of the current study warrant mention. First, it was not possible to determine the direct route of infection between femoral subcutaneous cysts and PJI after TKA. There are several reports that PJI occurs when there is an infection focused in the affected limb after TKA and that the infection spreads from there [17]. However, there are no reports of infection spreading from femoral subcutaneous cyst infection to TKA or any reports discussing this mechanism. It is possible that lower limb edema contributed to the worsening of the infection, but further clarification is needed.

Secondly, the origin of femoral subcutaneous cysts is unclear. Anatomically, the lymph nodes are distributed throughout the groin. The cyst was located slightly distal to the inguinal region and medial to the thigh region. This does not correspond to the anatomical location of the lymph nodes. Although the location of the subcutaneous cyst was consistent with the course of the lymphatic system of the lower extremity, it was suggested that the cyst originated from a part of it, which is not definitive. A relationship between the development of medial subcutaneous cysts on the thigh and *Echinococcus* infection was reported in a previous study [18]. However, the *Echinococcus* antigen test result was negative. Therefore, echinococcal infection was ruled out as the cause of the cyst formation in this case.

Finally, we did not perform a surgical resection of the subcutaneous cysts. The cyst regressed, and the pain disappeared after administration of antimicrobial agents. However, as previously reported, the cyst is considered to be at risk of infection. Therefore, if infection recurs in the future, we consider it necessary to resect the cyst.

## 4. Conclusion

The presence of subcutaneous cysts and their associated infection in the operated lower extremities after TKA are risk factors for PJI. The cause of a subcutaneous cyst infection and its spillover is lower extremity edema. Moreover, it is closely related to conditions that cause a loss of motion in the lower extremities, such as calcaneal fractures. Orthopedic surgeons should be aware of these symptoms when performing TKA. For example, the appearance of a subcutaneous cyst with infection or the development of a calcaneal fracture may cause lower extremity edema after TKA.

## Figures and Tables

**Table 1:** Laboratory data and events of the perioperative period.

	**Pre-OPE**	**Day 0**	**PO 1 week**	**PO 2 weeks**	**PO 6 weeks**	**PO 7 weeks**	**PO 9 weeks**	**PO 12 weeks**	**PO 12.5 weeks**
Events		L-TKA			Subcutaneous induration appears on the medial side of the thigh, started oral antimicrobial therapy(LVFX)	Discontinuing antibiotic	Calcaneal fracture, exacerbation of edema	Negative bacterial culture of knee joint fluid	Alpha-defensin test is positive, DAIR
WBC (∗10^3^/*μ*L)	4390	4340	4750	4160	5750	4960	6990	6840	6350
NEUT (%)	57.1	48.7	56.3	53.2	63.3	60.3	69.3	67.2	68.3
LYMP (%)	28.8	39.4	28.3	32.3	22.7	25.1	18.4	19.8	19.5
MONO (%)	8.4	6.7	8.8	8.8	9.3	9.7	8.8	9.7	9.6
EOS (%)	4.6	4.4	5.7	4.8	4	4.2	2.9	3	2.3
BASO (%)	1.1	0.9	0.9	0.8	0.6	0.6	0.6	0.4	0.3
CRP (mg/dL)	0.06	0.07	2.05	0.38	2.9	0.5	1	3.1	2.9
ESR (mm/h)	1.3	1.3	5.9	6	29	29	47	66	70

## Data Availability

The data supporting the findings of this study is available from the corresponding author, N.O., upon reasonable request.
